# Biomarkers for Circadian Rhythm Disruption Independent of Time of Day

**DOI:** 10.1371/journal.pone.0127075

**Published:** 2015-05-18

**Authors:** Kirsten C. G. Van Dycke, Jeroen L. A. Pennings, Conny T. M. van Oostrom, Linda W. M. van Kerkhof, Harry van Steeg, Gijsbertus T. J. van der Horst, Wendy Rodenburg

**Affiliations:** 1 Centre for Health Protection, National Institute for Public Health and the Environment (RIVM), Bilthoven, The Netherlands; 2 Department of Genetics, Center for Biomedical Genetics, Erasmus University Medical Center, Rotterdam, The Netherlands; 3 Department of Human Genetics, Leiden University Medical Center, Leiden, The Netherlands; McGill University, CANADA

## Abstract

Frequent shift work causes disruption of the circadian rhythm and might on the long-term result in increased health risk. Current biomarkers evaluating the presence of circadian rhythm disturbance (CRD), including melatonin, cortisol and body temperature, require 24-hr (“around the clock”) measurements, which is tedious. Therefore, these markers are not eligible to be used in large-scale (human) studies. The aim of the present study was to identify universal biomarkers for CRD independent of time of day using a transcriptomics approach. Female FVB mice were exposed to six shifts in a clockwise (CW) and counterclockwise (CCW) CRD protocol and sacrificed at baseline and after 1 shift, 6 shifts, 5 days recovery and 14 days recovery, respectively. At six time-points during the day, livers were collected for mRNA microarray analysis. Using a classification approach, we identified a set of biomarkers able to classify samples into either CRD or non-disrupted based on the hepatic gene expression. Furthermore, we identified differentially expressed genes 14 days after the last shift compared to baseline for both CRD protocols. Non-circadian genes differentially expressed upon both CW and CCW protocol were considered useful, universal markers for CRD. One candidate marker i.e. CD36 was evaluated in serum samples of the CRD animals versus controls. These biomarkers might be useful to measure CRD and can be used later on for monitoring the effectiveness of intervention strategies aiming to prevent or minimize chronic adverse health effects.

## Introduction

Human behavior, physiology and metabolism are subject to daily rhythms, which are controlled by the circadian clock. This endogenous time keeping system provides a temporal organization of our body functions in relation to environmental time and allows us to anticipate to daily recurring events [1]. Chronic circadian rhythm disruption (CRD), as encountered by frequent night shift work or multi time zone travelling might result in an increased risk for long-term health effects. Indeed, epidemiological studies among shift workers and flight personnel have associated frequent shift work and jet lag with an increased incidence of breast cancer, obesity and metabolic syndrome [2–4]. These adverse health effects occur after many years of shift work, and at present it is unclear what mechanism is causing adverse health effects and how these effects of shift work can be minimized. The ability to measure chronic CRD associated with shift work would allow measuring effects of interventions on chronic CRD and monitoring adversity in shift workers and ultimately will help to design intervention strategies.

Studies on the beneficial effects of interventions to prevent shift work-driven adverse health outcomes assess effects on CRD using classical circadian markers, including melatonin, cortisol and body temperature [5]. These markers allow monitoring circadian rhythm and acute CRD using multiple measurements around the clock before health effects occur. In addition to classical circadian markers, recent research on circadian clock controlled output genes has shown that up to 10% of the transcribed genes is under circadian control, providing additional rhythmic markers to estimate body time in blood and tissues [6, 7]. However, both the classical circadian markers and cycling clock and clock-controlled gene markers are non-eligible as CRD markers in large-scale human cohort studies due to two important pitfalls. Firstly, circadian markers require around the clock measurements, resulting in higher costs and larger impact on participating subjects compared to single measurements. Secondly, classical biomarkers are useful for demonstrating acute CRD, but provide no or only limited information on long-term CRD and accumulation of adversity over time. To acquire information on biological adversity of CRD and to explore the effectiveness of CRD preventive measures, new biomarkers are needed to evaluate the presence of chronic CRD in a time of day independent manner.

Shift work involves a multitude of aspects, including phase desynchronization, light at night, sleep disruption and lifestyle disturbances, all of which potentially play a role in causing CRD and associated adverse health effects [8]. Many different shift work schedules are in use, varying in rotation speed and direction, including forward (counterclockwise) or backward (clockwise) rotating shift schedules. Experimental studies in which mice were subjected to (chronic) shifts in the light-dark cycle (as such resembling jet lag), have shown that both counterclockwise (CCW) and clockwise (CW) schedules cause CRD [9]. Additionally, several human studies have shown disturbed circadian rhythms by both CCW and CW work schedules, without major differences between the schedules [10–12]. However, in aged mice CCW shifts appeared more disruptive than CW shifts, as evident from the increased mortality [13].

The aim of the present study was to identify universal biomarkers for CRD independent of rotation direction and time of day. Two different rotations of chronic jet lag were used to induce CRD. Since blood biomarker discovery is technically challenging, we selected the liver to identify biomarkers, as the target tissue of metabolic effects of CRD and as previously used for circadian transcriptomics studies [7]. By comparing the liver transcriptome of animals under normal, CW rotating and CCW rotating light schedules, we identified a set of hepatic gene expression markers that report on the presence of CRD. Additionally, we identified non-circadian, age-independent genes differentially expressed after CRD compared to baseline that are potentially blood detectable. One candidate biomarker *i*.*e*. CD36 was validated in blood, allowing future use in large-scale human studies.

## Methods

### Study design

Animal studies were performed in compliance with national legislation, including the 1997 Dutch Act on Animal Experimentation, and experiments were approved by the Animal Experimentation Ethical Committee of the National Institute for Public Health and the Environment in Bilthoven. All surgery was performed under isoflurane anesthesia and appropriate analgesics were used to minimize suffering.

Female FVB mice, 8 to 12 weeks of age, were kept under a normal 12:12 hour light-dark (LD) cycle for approximately three weeks, with Zeitgeber Time 0 (ZT0) corresponding to lights on. Animals were group-housed and food and water were provided *ad libitum*. Prior to the first shift, animals (n = 24) were sacrificed around the clock at four hour intervals (n = 4 at each time point). Subsequently, the remaining group of animals underwent six shifts in either a clockwise (CW) or counterclockwise (CCW) rotating light schedule. Specifically, mice were exposed to a phase delay or phase advance of eight hours every five days, respectively (S1 Fig). Hereafter, mice were again kept under LD, referred to as recovery. After one shift, six shifts, five days recovery and fourteen days recovery n = 30 animals per group were sacrificed around the clock with four hour intervals (n = 5 per time point) by orbital bleeding under Ketamine/Xylazine anesthesia. Liver and blood were collected for transcriptomics and serum analyses, respectively. A detailed overview of the experimental design is depicted in S1 Fig.

Four additional mice per group received a radio transmitter (Physio Tel, TA11 TA-F10; Data Sciences, St. Paul, MN) in the peritoneal cavity to record core body temperature every ten minutes. Body temperature was recorded throughout the experiment. Cosine curves were fitted using the R statistical software environment (www.r-project.org) to determine the phase (i.e., peak time) of activity and body temperature rhythms.

### Microarray analysis

RNA was extracted from livers (n = 4 per time point at baseline and n = 2 per time point for the CCW and CW groups) using the miRNeasy Mini Kit (Qiagen Benelux, Venlo, The Netherlands). RNA concentrations were measured using a NanoDrop ND-1000 Spectrophotometer (Nanodrop Technologies, Wilmington, DE, USA), and RNA quality was assessed with an Agilent 2100 Bioanalyzer (Agilent Technologies, Amstelveen, the Netherlands).

RNA was processed for gene expression analysis at the Microarray Department of the University of Amsterdam, the Netherlands, using methods described in Pennings *et al* [14]. Experimental samples (each corresponding to RNA from one individual mouse) were labelled with Cy3 and the common reference sample (made by pooling equimolar amounts of RNA from experimental samples) was labelled with Cy5. Samples were hybridized to Nimblegen Mus musculus 12 x 135 k microarrays (Roche NimbleGen, Germany). This type of microarray contains 44,170 gene probes with three spots per probe. Slides were scanned with an Agilent G2565CA DNA microarray scanner. Feature extraction was performed with NimbleScan v2.5 (Roche NimbleGen), resulting in a table containing individual probe signal intensities for both dyes.

The raw data were subjected to a set of quality control checks to ensure comparable signal average and distribution. Raw microarray data for gene-coding probes were normalized in R (www.r-project.org) using a four step approach [14]: (1) natural log-transformation, (2) quantile normalization of all scans, (3) correcting the sample spot signal for the corresponding reference spot signal and (4) averaging data from replicate probe spots. The normalized data of 44,170 probes were further analysed in R and Excel (Microsoft Corporation, USA). Probe to gene annotation data was downloaded from NCBI. Classification and statistical analysis was performed on the probe level. Significantly predictive or regulated probe sets were annotated to the corresponding genes for further biological interpretation. To this end, when multiple probes corresponding to the same gene were significant, they were counted as one gene in further analysis; probes that did not correspond to genes according to the current NCBI database were excluded from further analysis.

Complete raw and normalized microarray data and their MIAME compliant metadata have been deposited at GEO (www.ncbi.nlm.nih.gov/geo) under accession number GSE65346.

### Classification approach

After normalization, a classification approach was applied to identify a set of genes able to classify samples into either circadian rhythm disrupted (CRD) or non-disrupted (ND) 14 days after the last shift. Three different algorithms were used: Random forests (RF) [15], Support vector machine (SVM) [16] and Prediction analysis for Microarrays in R (PAM-R) [17] (R statistical software). To ensure time-of-day independence of the classifier, for each classification algorithm, a ‘leave-one-time point-out’ cross-validation approach was used for the classification. Here, time point refers to ZT time point. To this end, the data were repeatedly split into a training set and a test set, in which the training set comprised the data for all-but-one time point, and the test set the data for the remaining time point. The prediction model obtained for the training set was used to predict the test set data. This approach keeps replicate (time point) samples together and therefore gives a more reliable estimation of the prediction accuracy than cross-validation with random sample selection. The prediction accuracy was calculated as the average over all the test set predictions. As each classifier builds a different prediction model (potentially using different genes) for each training set, genes included in the majority of the models for each classifier were taken as consensus gene set for each type of classifier.

As we aim to find biomarkers which applicability does not depend on a specific choice of algorithm, only genes present in all three classifiers consensus sets were considered potentially valuable biomarkers to classify CRD versus non-disrupted [18, 19]. To validate prediction accuracy of the consensus gene set after one shift, six shifts and after 5 days recovery, the 14 days recovery dataset was used as the training set to build the prediction model.

### Sequential approach

To identify differentially expressed genes (as compared to baseline) after 14 days recovery for the CCW and CW protocol separately, we performed a one-way ANOVA with Qlucore Omics Explorer (Qlucore AB, Lund, Sweden) in which p<0.001 was considered statistically significant. CircWave Batch v5.0 software (Hut, R., www.euclock.org/results/item/circ-wave-batch.html) was used to analyze circadian rhythmicity of gene expression. P-Values were false discovery rate (FDR) corrected [20]; genes with an FDR <0.05 were considered rhythmically expressed. Genes that were rhythmically expressed at any time point during the experiment (baseline, 1 shift, 6 shifts 5 or 14 days recovery) were excluded as potential biomarker. The GenAge Database of ageing-related genes (www.genomics.senescence.info) was used to identify (human) age-dependent genes, which were excluded also.

To determine which candidate biomarker genes are potentially detectable in human serum or plasma, we determined which genes code for proteins that are annotated in Gene Ontology as extracellular or in UniProt as secreted. Additionally, we determined which genes have human equivalents that have been experimentally detected with high confidence in plasma or serum [21] or as part of the Human Plasma Proteome Project [22].

### Biomarker serum levels

Corticosterone serum levels were determined using ELISA assays (Yanaihara Institute Inc. Shizuaka, Japan) and subsequently visualized and analysed using GraphPad Prism software version 6.04 for Windows (GraphPad Software, San Diego California USA). Five outliers (out of 251 samples) were excluded based on Grubbs analysis (alpha 0.1). Phase and circadian rhythm were analysed using CircWave Batch v5.0 software. CD36 was determined in serum with a dedicated ELISA assay (Abcam, Cambridge, United Kingdom). Differences between CRD exposed groups and baseline were statistically tested using a two-sided student’s t test. P-values <0.05 were considered statistically significant.

## Results

### Classical circadian markers

**Fig 1:**
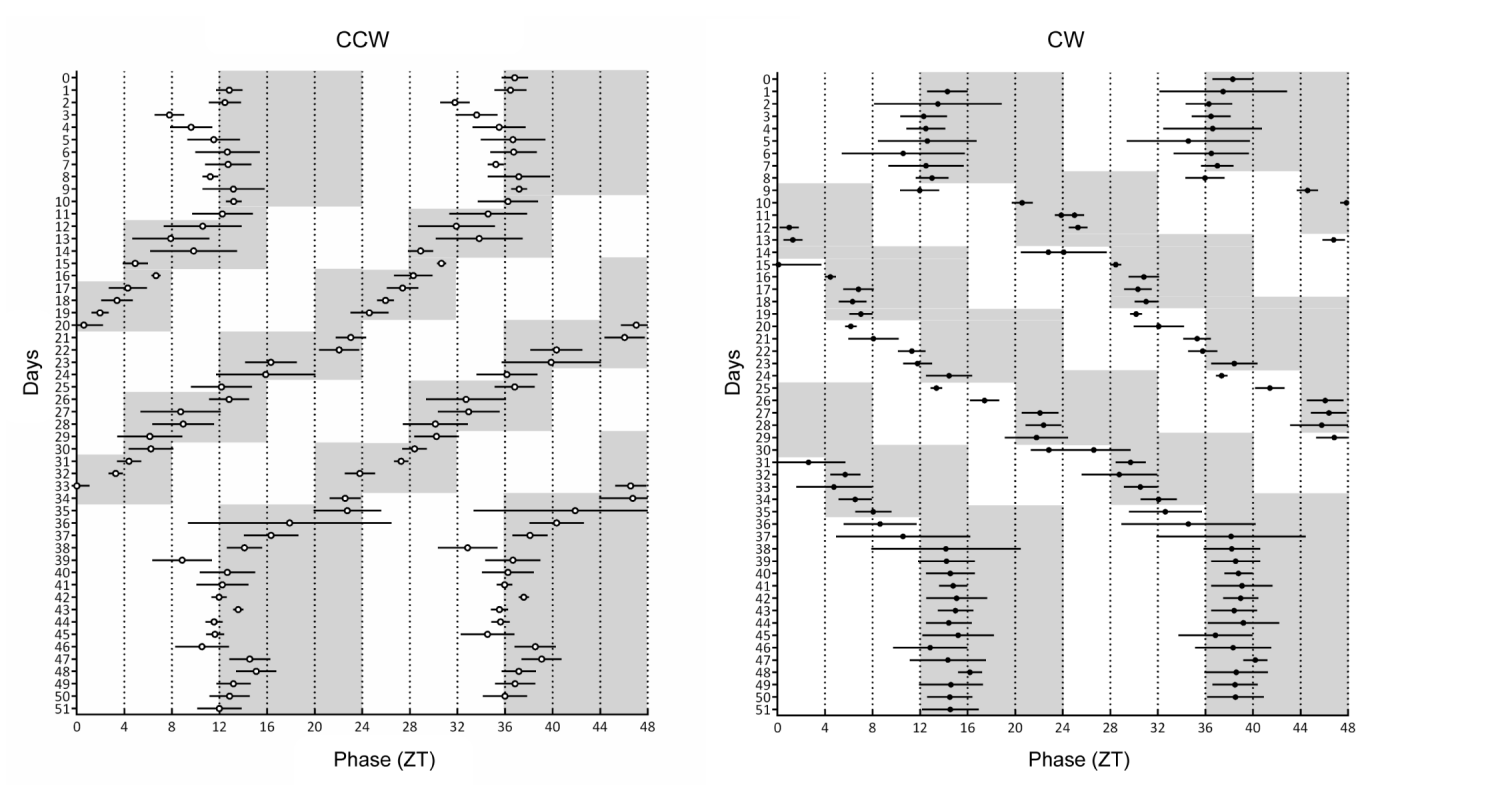
Double plots of peak phase of core body temperature rhythms. **A.** the counterclockwise schedule and **B.** the clockwise schedule. A cosine function was fitted to determine peak phase (n = 4 mice per group). Data are presented as mean peak phase ± sd. Grey areas indicate periods of darkness.

First, we evaluated whether the two different schedules, counterclockwise (CCW) and clockwise (CW), affected circadian rhythm by analyzing classical circadian markers: core body temperature and corticosterone rhythms. At baseline, animals showed regular daily body temperature rhythms, with peaks at approximately ZT12. Core body temperature rhythms re-entrain to the new light-dark cycle after the first shift and following shifts, for both CCW and CW groups (Fig 1, panels A & B, respectively).

**Fig 2:**
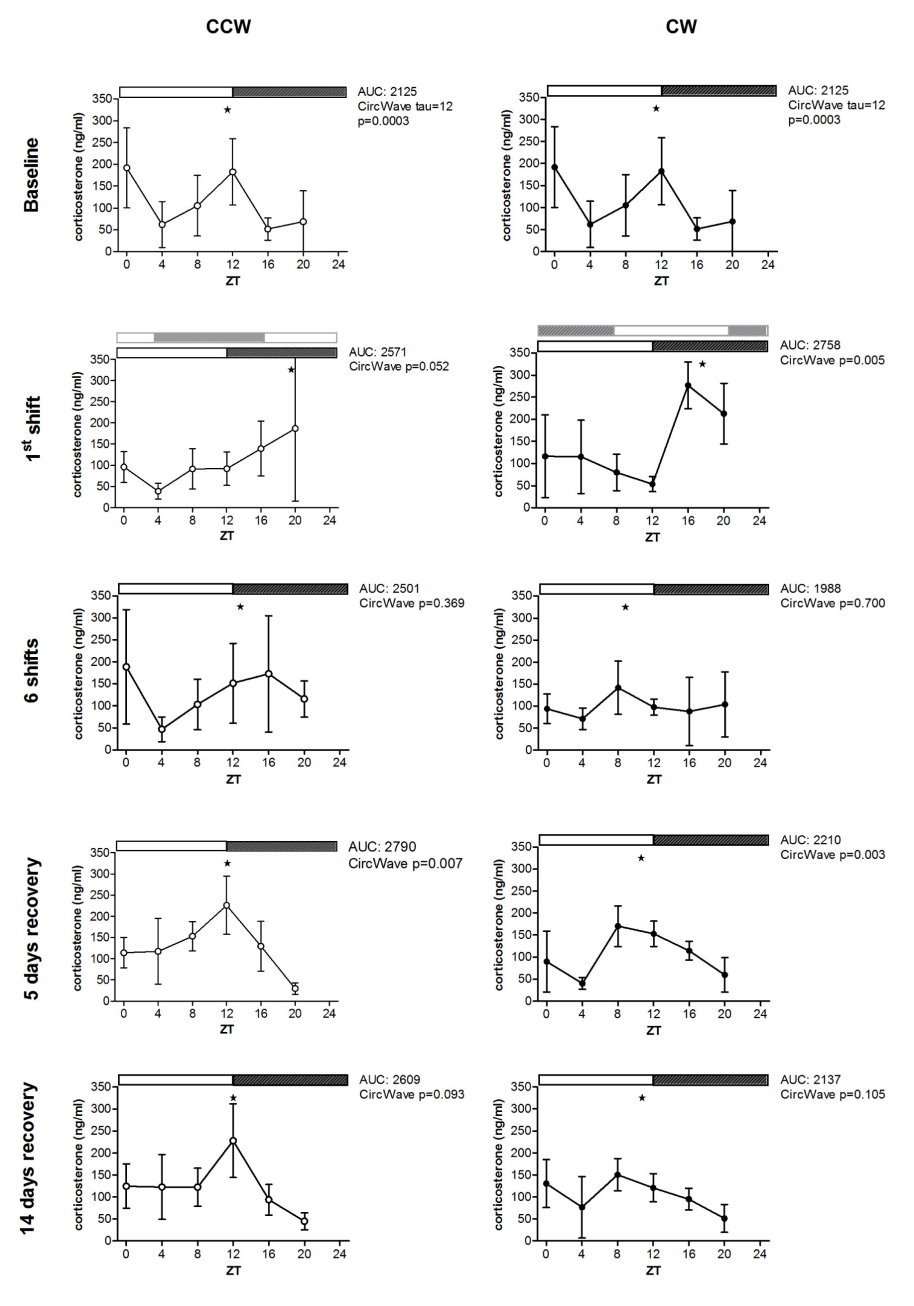
Corticosterone serum levels for the counterclockwise (CCW) and clockwise (CW) jet lag protocols. Serum levels were determined at baseline, after 1 shift, 6 shifts, 5 days recovery and 14 days recovery. Data are presented as mean ± SD. For the first shift, the light dark schedule before the shift is depicted in grey. At baseline a significant 12-hr rhythm was detected, due to the major peak at ZT12 and another peak at ZT0. CircWave peak phases were indicated with (★). Note: as circadian rhythmicity of serum corticosterone levels was lost after 6 shifts, peak phases at the 6^th^ shift are just indicative of the best cosine fit.

As expected, corticosterone serum levels at baseline showed a major peak at ZT12. Additionally, a minor peak at ZT0 was also observed, corresponding with a small increase in activity at this time point specific for FVB mice (CircWave tau = 12, *p* = 0.0003). Differential effects between the two schedules could be observed after peak phase analysis of corticosterone rhythms (Fig 2). After the first shift, the corticosterone rhythm shows peak levels at ZT16 for the CW group, representing an incomplete shift (*p* = 0.005). In the CCW group peak time was at ZT20, indicative of a lack of phase shift at this time point (*p* = 0.052). Although there is a tendency toward circadian rhythm, no significant circadian corticosterone rhythm could be detected in either group upon six shifts (CCW: *p* = 0.369, CW: *p* = 0.700). At 5 days and 14 days after the last shift, rhythms were detected in both groups. After 14 days recovery these rhythms were less robust for CCW and CW (*p* = 0.093 and *p* = 0.105, respectively) compared to 5 days after the last shift (CCW: *p* = 0.007, CW: *p* = 0.003). Overall, these results show that circadian rhythms of corticosterone levels heavily disturbed upon prolonged exposure to CRD, but reappear when animals are back under normal LD conditions.

In summary, both experimental jet lag schedules, CCW and CW rotation affected the classical circadian markers, indicating CRD. Minor differences in classical circadian markers were found between the two schedules directly after a shift. However, for both schedules the effects were transient, largely recovering within 14 days after the last shift.

### Predictive set of hepatic transcriptome markers

**Fig 3:**
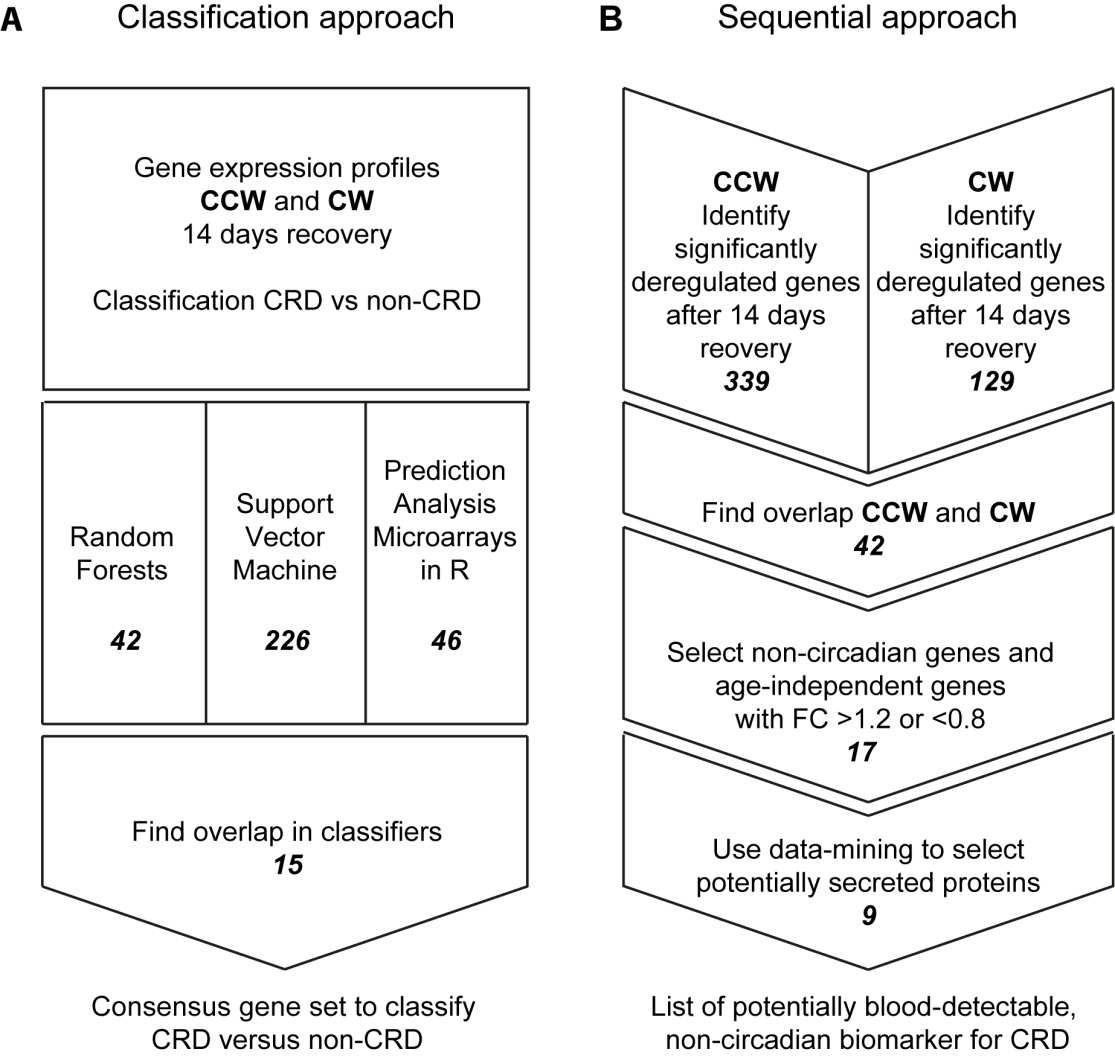
Schematic overview of the microarray analyses. **A.** Overview of the classification approach, resulting in a *set* of transcriptomics biomarkers including 15 individual genes. **B.** Outline of the sequential approach of the data-analyses from hepatic gene expression profiles to 9 potential blood-detectable protein biomarkers.

Since liver is the target tissue of metabolic effects and can be used for future studies, we performed an around the clock analysis of the liver transcriptome at baseline and after 14 days recovery. To identify a predictive set of hepatic transcriptome markers for chronic CRD with time of day-independent expression levels, we applied a classification approach. Three different classification algorithms were used: RF, SVM and PAM-R (complete overview in Fig 3a). Using a ‘leave-one-time point-out’ cross-validation approach, we identified one consensus set of genes per algorithm optimally classifying samples as CRD versus non-disrupted, after 14 days recovery independent of time of day. The SVM approach resulted in a consensus set of 226 probes, RF in 42 and PAM-R in 46. Only the 18 probes, corresponding to 15 individual genes, present in all three classifiers were considered potentially robust biomarkers to classify CRD versus non-disrupted [18, 19] (Table 1, S2 Fig). Based on this consensus gene set prediction accuracy was achieved ranging from 90% to 98% (S1 Table) showing that the set of 15 genes could distinguish CRD-exposed animals from non-disrupted controls with high accuracy independent of sampling time.

**Table 1:** Potential biomarkers for CRD as identified by classification and sequential approach.

Gene symbol	Function	Consensus gene set (classification approach)	Non-invasive blood detectable marker (sequential approach)
*Cyp2c29*	Cytochrome P450 epoxygenase	x	X
*Cyp2b10*	Cytochrome P450		X
*Rbp1*	Vitamin A transport	x	X
*Sult2a1*	Sulfotransferase	x	
*Cd36*	Scavenger receptor	x	x
*Ntrk2*	Kinase signaling	x	x
*Tusc3*	Tumor suppression	x	
*Armcx3*	Tumor suppression	x	
*Gspt2*	Cell cycle progression	x	
*Snrpn*	Transcription	x	x
*Tceal8*	Transcription	x	
*Fkbp11*	Protein folding	x	
*Orm2*	Acute phase plasma protein	x	
*Gm3787*	Unknown	x	
*Gm9299*	Unknown	x	
*D630033O11Rik*	Unknown	x	
*Igh-VJ558*	Immune		x
*Srgap3*	GTPase activity		x
*Tram1*	Translocation proteins		x

Subsequently, the ability of the consensus gene set to detect CRD after one shift, 6 shifts and 5 days recovery was determined. Detection of acute CRD was limited, as after the first shift only 29% to 33% of the samples of phase shifted animals were correctly classified as CRD samples, depending on classification method. Accumulation of CRD was detected in the samples taken after six shifts, here, 92% to 88% of the samples were correctly classified as CRD exposed. Samples taken five days after the last shift were also well classified by the gene set, 75% to 88% depending on the algorithm (S2 Table). Overall, this hepatic transcriptome marker set is well able to detect chronic CRD independent of the time point the sample is collected within 14 days recovery.

### Non-invasive biomarkers for CRD

Although predictive of CRD, use of hepatic gene expression markers is still invasive, where non-invasive methods are preferable. However, direct biomarker discovery in blood is technically challenging. To find non-invasive biomarkers eligible to monitor CRD, we aimed to identify potential blood markers for CRD from the gene expression dataset. Therefore, we selected genes of which expression (i) increased or decreased with accumulating CRD exposure and (ii) remained deregulated after 14 days recovery. Furthermore, we excluded genes with circadian expression levels as these have drawbacks earlier described. Compared to control animals, 339 genes were differentially expressed in mice exposed to the CCW schedule. For mice in the CW rotation schedule (*p* <0.001) 129 were differentially expressed, of which 42 genes were significantly expressed in both groups. Of these 42 genes, only genes with a fold change (FC) larger than 1.2 or smaller than 0.8 were considered relevant to increase the probability of detectable differences in blood protein levels. This resulted in 17 genes all showing an approximate pattern of up or down regulation with accumulating shifts and remaining differentially expressed after 14 days recovery compared to baseline.

**Fig 4:**
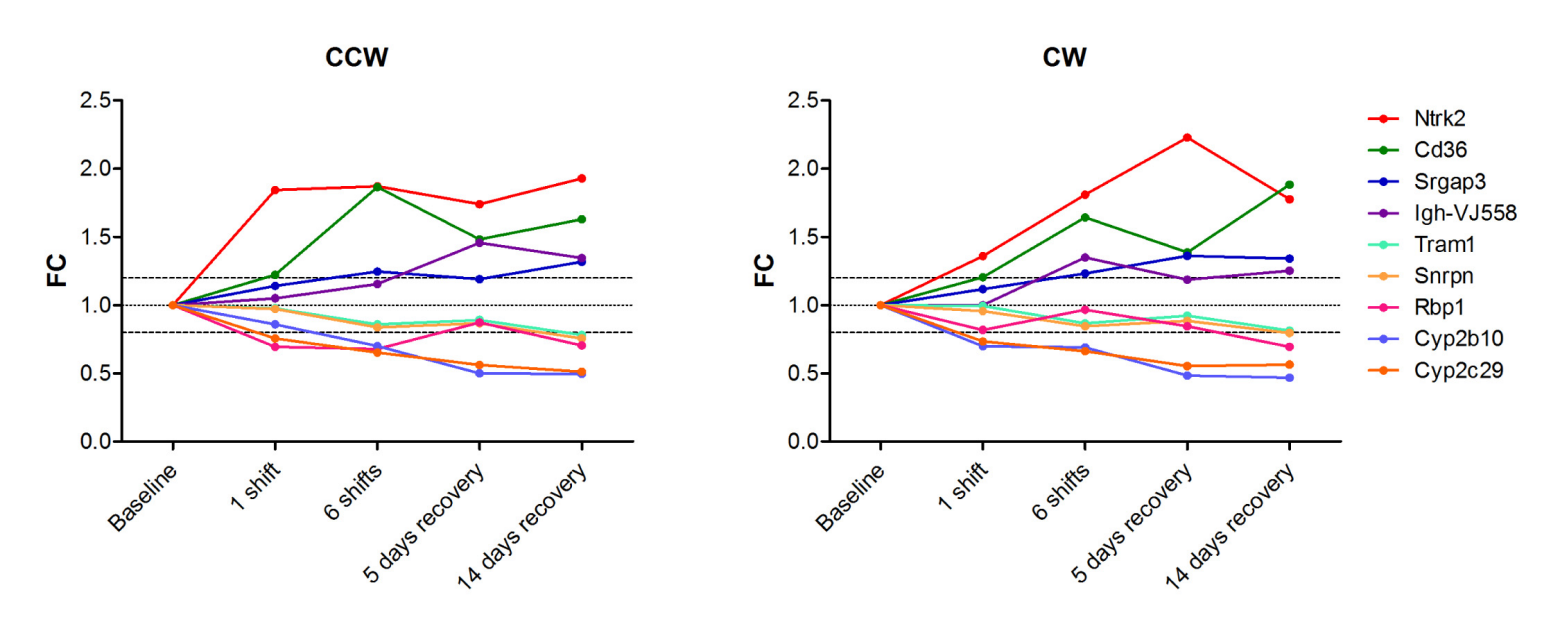
Potentially blood-detectable marker for CRD. Fold ratio of selected differentially expressed genes encoding potentially blood-detectable protein biomarkers for CRD. Expression of these genes is up-regulated or down-regulated by both the CCW and the CW schedule and remains up or down regulated during recovery.

Of these 17 genes, 9 genes were found to encode potentially blood-detectable biomarkers according to (i) annotation as “secreted” or “extracellular”, and/or (ii) previous proteome-based experimental detection in human plasma or serum: *Cd36*, *Ntrk2*, *Igh-VJ558*, *Srgap3*, *Tram1*, *Snrpn*, *Rbp1*, *Cyp2b10* and *Cyp2c29* (see Fig 3b and S3 Table for the sequential flow of gene selection). A substantial overlap with the classification consensus gene set was found, 5 genes were identified by both approaches (Table 1). Four out of the 9 genes showed increasing up regulation with number of shifts and remained up-regulated during recovery and five genes showed a similar pattern in the opposite direction (Fig 4).

**Fig 5:**
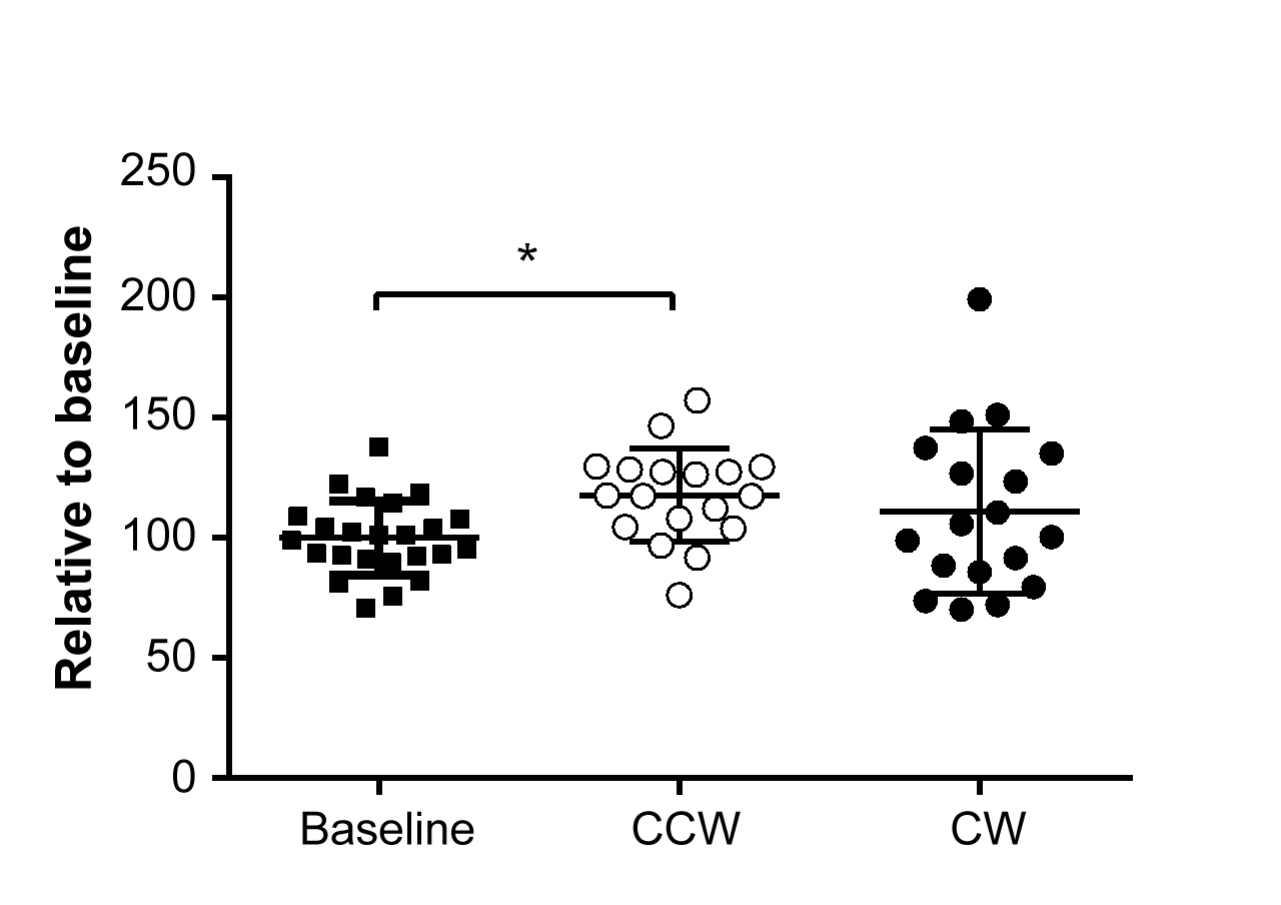
Serum levels of the CD36 protein. Serum levels were determined at baseline and 14 days after the last shift for animals subjected to CCW and CW shifted light schedules. Error bars indicate mean ± sd.

Based on expression patterns and the availability of a serum ELISA assay, CD36 was selected for validation in blood. At 14 days after the last shift, CD36 serum levels showed a significant increase of 18% in animals exposed to CCW shifted light schedules independent of time of day, compared to animals at baseline (Fig 5). For the animals exposed to the CW light schedule, an 11% increase was found. For both shifting schedules, the increase in blood levels was less pronounced than the increase observed in the gene expression data.

## Discussion

Frequent shift work results in a disruption of the circadian rhythm (CRD) and might on the long-term result in increased health risk. Epidemiologic studies among shift workers and flight personnel have shown increased risk of breast cancer, obesity and metabolic syndrome [2–4]. The growing 24/7 economy will only lead to increase in shift work and consequently will adversely affect health. Ideally, the level of chronic CRD should be detected before the negative health effects occur. To limit potential health effects, preventive measures to minimize CRD are an attractive option. For these purposes, measuring the presence of chronic CRD is of importance, however, tools to measure chronic CRD are currently lacking. The present study aimed to identify universal biomarkers for chronic CRD using a transcriptomics approach.

To date, CRD and the beneficial effects of preventive measures on CRD are mainly determined using classical circadian markers such as melatonin, cortisol or body temperature [23]. There is a need of markers that show the cumulative effects of CRD, since the effects on available markers are transient showing only acute effects of CRD. Corticosterone rhythms were heavily disturbed after six subsequent shifts of the light dark schedule and reappear after 5 days recovery, whereas body temperature remains rhythmic and was found to re-entrain to the new light dark schedule within 4–5 days, even after multiple shifts. The present study identified chronic, non-transient biomarkers for chronic CRD 14 days after the last shift, based on hepatic gene expression. The potentially blood detectable biomarkers report on the presence of chronic CRD, showing an increase or decrease after multiple shifts and non-reversible deregulation upon recovery. The classification markers (consensus gene set) could be used directly after multiple shifts and 5 or 14 days after the last shift, which makes good candidates to determine acute shift work related CRD.

Ideally, for large-scale molecular epidemiology and experimental studies biomarkers of CRD are time-independent. The current available circadian markers require 24-hr measurements, which is challenging in both experimental and field studies. Recent attempts have been made to identify transcriptomics- and metabolomics-based time of day independent biomarkers. In these studies, jet lag and clock mutant mice were used [7, 24], allowing detection of acute and transient effects of jet lag but not chronic effects. It is challenging to model human shift work since it involves a multitude of aspects, including phase desynchronisation, changed social patterns, activity, sleep, nutrition light exposure and sun exposure. Using a chronic jet lag model in mice, and as such mainly mimicking phase desynchronisation, we were able to identify biomarkers detecting chronic CRD that can be studied in a single sample, collection of which is independent of time of day. Further studies should point out whether other aspects of shift work (e.g. changes in activity, light-at-night, altered nutritional timing) will affect the same genes.

The set of liver-transcriptome based biomarkers includes genes with a variety of functions. The current study suggests that CD36 could potentially be a blood-detectable biomarker for CRD. CD36 is a scavenger receptor present on many mammalian cell types with a broad range of cellular functions [25]. It has been suggested that CD36 in plasma might represent a marker of the metabolic syndrome [26], a condition that was found associated with frequent shift work [27]. Furthermore, CD36 was shown to play an important role in breast tumorigenesis [28, 29] potentially associated with the observed increase in breast cancer risk found in shift workers [30]. Although CD36 is interesting as CRD biomarker, other potentially blood-detectable biomarkers should not be neglected. Especially, the genes that were found upregulated upon CRD are of interest for further investigation. For example, *Ntrk2* shows a similar induction pattern as CD36 and has also been shown to play a role in breast cancer cell survival and obesity [31, 32]. In depth analysis of biological relevant data in this study is limited due to our aim to select non-circadian biomarkers, for which circadian genes were excluded. Full analysis of biological processes affected by CRD requires inclusion of these circadian genes and is subject for further studies.

Our study represents two approaches to identify the most valuable CRD markers based on hepatic gene expression; firstly, an optimal CRD classification set and secondly, a selection of potentially blood detectable biomarkers. An important step that needs to be taken before the identified biomarkers can be applied in experimental and large-scale cohort studies is validation for CRD in humans. For blood-detectable markers, the challenge of the transcriptomics approach is the translation of hepatic gene expression to protein levels in blood. We found that in our homogenous mouse model, CD36 serum levels were also increased in animals exposed to CCW and CW shifted light schedules; however, the increase was smaller compared to gene expression. Potentially, serum CD36 originates from other sources than liver alone, since CD36 is present in many mammalian cell types [25]. Another part of the validation process is the exclusion of post-translational rhythmicity, which is not precluded by the lack of a transcriptional rhythm. In human samples, heterogeneity and variation between samples is much larger and small increases in biomarker blood levels may remain undetected in cross-sectional studies. Preferably, to obtain less inter-individual variation one would opt for longitudinal measurements including baseline measurements per individual before commencing shift work rotations. Furthermore, anchoring with phenotypic endpoint is required to use the selected biomarkers for intervention studies without the need for long-term end-point studies. For this, it should be noted that previous studies have shown that comparable chronic CRD protocols increased negative health effects in mice [13, 33].

In conclusion, our study identified a chronic CRD gene-set, comprising 15 genes, potentially useful to study CRD induction by different aspects of shift work and reduction by interventions. Furthermore, we identified 9 candidate genes for blood-detectable biomarkers of CRD, including CD36. Upon validation, these biomarkers provide valuable tools for evaluating CRD in both experimental animal and human studies set up to identify preventive measures for adverse health effects.

## Supporting Information

S1 Fig Schematic overview of the experimental set up and time of sample collection. White areas indicate lights on, grey areas indicate lights off.
(TIF)

S2 Fig Venn-diagram of selected hepatic gene expression biomarkers using the three classifier algorithms. The consensus gene set consists of the 15 genes overlapping between the three algorithms.
(TIF)

S1 Table Results of prediction of the three classifier algorithms, using the consensus gene set (A-C). A summary of the prediction accuracy and other prediction parameters is shown in (D).
(DOCX)

S2 Table Ability of the consensus gene set to correctly classify phase-shifted animals as CRD after one shift, six shifts or five days recovery using the three classifier algorithms. (DOCX)

S3 Table Sequential approach gene selection. (XLSX)
