# Supplementary figures and images for: Biomarkers for Circadian Rhythm Disruption Independent of Time of Day
Source: PLoS One. 2015 May 18;10(5):e0127075. doi: 10.1371/journal.pone.0127075 (PMC4436131; doi:10.1371/journal.pone.0127075)

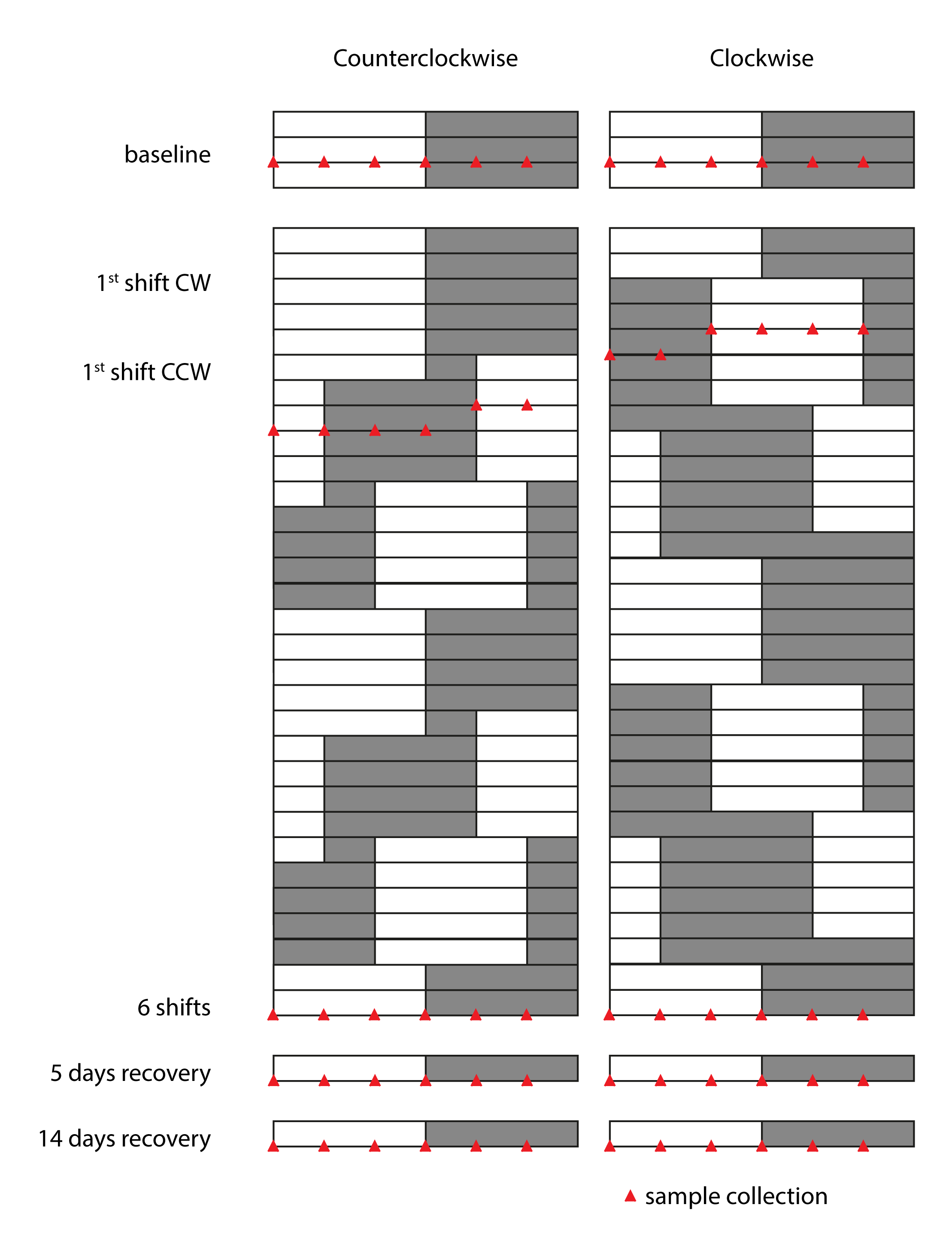

Supplement: S1 Fig — White areas indicate lights on, grey areas indicate lights off. (TIF) [file pone.0127075.s001.tif]

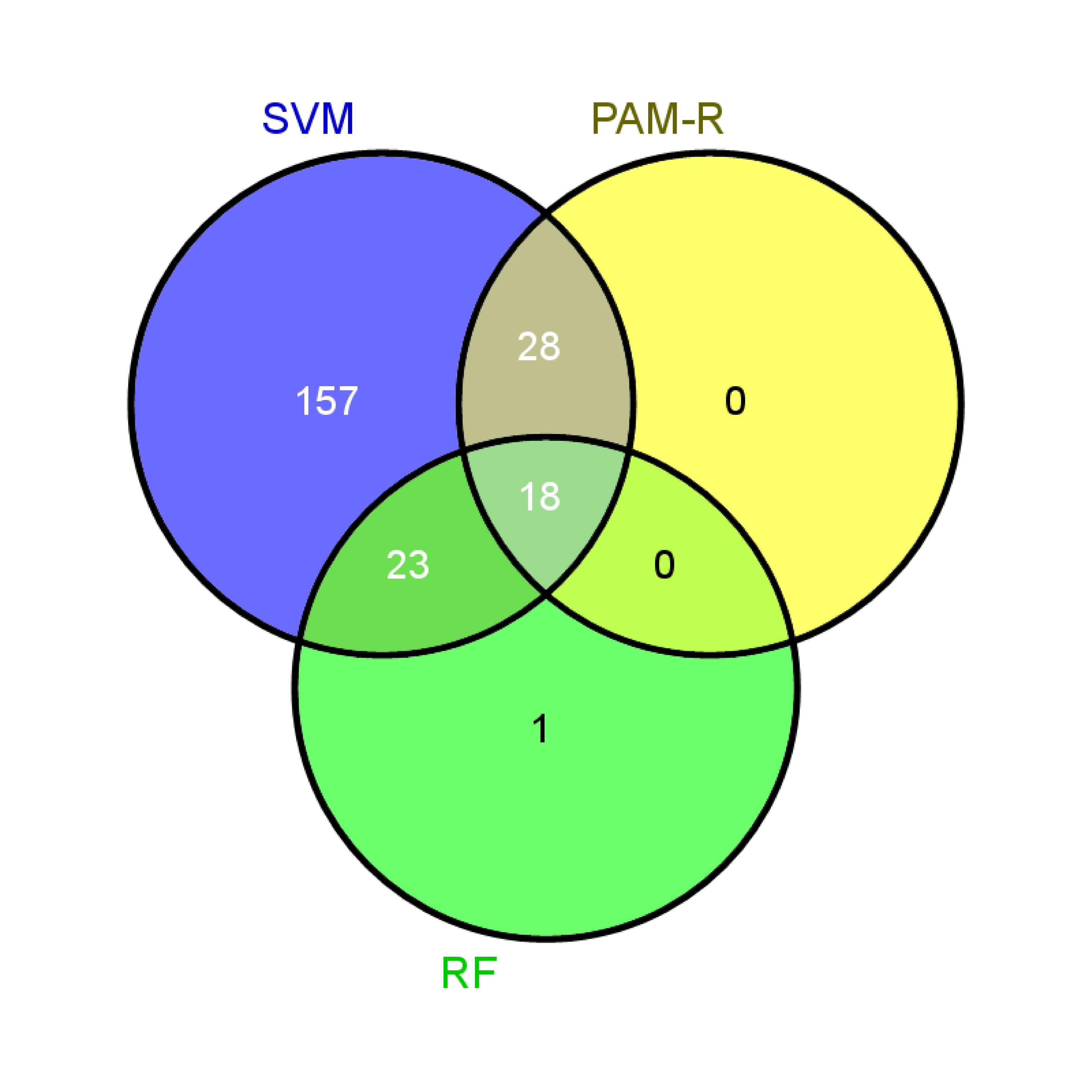

Supplement: S2 Fig — The consensus gene set consists of the 15 genes overlapping between the three algorithms. (TIF) [file pone.0127075.s002.tif]
