# Supplementary material for: Biomarkers for Circadian Rhythm Disruption Independent of Time of Day
Source: PLoS One. 2015 May 18;10(5):e0127075. doi: 10.1371/journal.pone.0127075 (PMC4436131; doi:10.1371/journal.pone.0127075)
Supplement: S1 Table — A summary of the prediction accuracy and other prediction parameters is shown in (D). (DOCX) [file pone.0127075.s003.docx]

| **A.** |  | **SVM** | |  |
| --- | --- | --- | --- | --- |
|  |  | **Non-CRD** | **CRD** |  |
| **Experiment** | **Non-CRD** | 23 | 1 | **24** |
|  | **CRD** | 0 | 24 | **24** |
|  |  | **23** | **25** |  |

| **B.** |  | **PAM-R** | |  |
| --- | --- | --- | --- | --- |
|  |  | **Non-CRD** | **CRD** |  |
| **Experiment** | **Non-CRD** | 21 | 3 | **24** |
|  | **CRD** | 0 | 24 | **24** |
|  |  | **21** | **27** |  |

| **C.** |  | **RF** | |  |
| --- | --- | --- | --- | --- |
|  |  | **Non-CRD** | **CRD** |  |
| **Experiment** | **Non-CRD** | 21 | 3 | **24** |
|  | **CRD** | 2 | 22 | **24** |
|  |  | **23** | **25** |  |

| **D.** | **SVM** | **PAM-R** | **RF** |
| --- | --- | --- | --- |
| **Accuracy** | 97.9% | 93.6% | 89.6% |
| **Sensitivity** | 100% | 100% | 91.7% |
| **Specificity** | 95.8% | 87.5% | 87.5% |
| **Positive predictive value** | 96.0% | 88.9% | 88.0% |
| **Negative predictive value** | 100% | 100% | 91.3% |
