# Supplementary material for: Biomarkers for Circadian Rhythm Disruption Independent of Time of Day
Source: PLoS One. 2015 May 18;10(5):e0127075. doi: 10.1371/journal.pone.0127075 (PMC4436131; doi:10.1371/journal.pone.0127075)
Supplement: S2 Table — (DOCX) [file pone.0127075.s004.docx]

|  | **Sensitivity** | | |
| --- | --- | --- | --- |
|  | **SVM** | **PAM-R** | **RF** |
| **1 shift** | 33.3% | 29.2% | 29.2% |
| **6 shifts** | 87.5% | 91.7% | 83.3% |
| **5 days recovery** | 83.3% | 87.5% | 75.0% |
